# Compensation effect of winter wheat grain yield reduction under straw mulching in wide-precision planting in the North China Plain

**DOI:** 10.1038/s41598-017-00391-6

**Published:** 2017-03-16

**Authors:** Xinhui Liu, Yujie Ren, Chao Gao, Zhenxing Yan, Quanqi Li

**Affiliations:** College of Water Conservancy and Civil Engineering, Shandong Agricultural University, Tai’an, 271018 China

## Abstract

Climate change and the growing demand for food security force growers to identify ways both to improve food production and to reduce agricultural carbon emissions. Although straw mulching is known to decrease CO_2_ emissions, winter wheat grain yield in the North China Plain was declined under straw mulching. In an effort to determine the most effective way to increase winter wheat yield under straw mulching, a field experiment was conducted using two planting patterns (wide-precision planting and conventional-cultivation planting) and two straw mulching rates (0 and 0.6 kg/m^2^). The results showed the wide-precision planting/non-mulching treatment significantly increased the leaf area index more than the other three treatments at the early growth stage. This treatment improved aboveground dry matter accumulation and was conducive to increased spike weight in the late growth stage. By contrast, straw mulching significantly reduced winter wheat grain yields by lowering both spike number and 1000-grain weight at the mature plant stage. In the wide-precision planting/mulching treatment, a significantly increased spike number compensated for grain yield losses. The results support the idea that wide-precision planting combined with straw mulching has the potential to decrease the winter wheat grain yield reduction previously observed with straw mulching in the North China Plain.

## Introduction

Wheat is the world's third most widely grown crop and an essential source of calories for millions of people; therefore, maintaining and increasing global wheat production is strongly linked to food security^1^. The North China Plain is the main wheat production area in China. In this region, a winter wheat and summer maize double cropping system is often adopted. The rapid expansion of manufacturing in the North China Plain has redirected the local water supply away from farming, resulting in agricultural water shortages in the North China Plain in recent years^2^. Moreover, water use efficiency in winter wheat fields is very low, because approximately one-third of the water is lost to soil evaporation^3^.

Agriculture also significantly contributes to the release of greenhouse gases, and its contribution to climate change is approximately 14% on an annual basis^4^. Small changes in the magnitude of soil respiration could have a large effect on the concentration of CO_2_ in the atmosphere^5^. Hence, strategies are required to reduce CO_2_ emissions^6^. Soil is the largest terrestrial organic carbon sink, and therefore, soil carbon sequestration is one way to offset atmospheric carbon dioxide enrichment^7^.

Straw mulching can help reduce soil water evaporation and CO_2_ emissions. Stagnari *et al.* ^8^ found that straw mulching could improve soil water retention. This effect reduces soil water evaporation losses and increases plant transpiration rates. Hari *et al.* ^9^ showed that when irrigation is limited, straw mulching increases wheat yield, water use efficiency, and soil organic carbon fixation. However, to date, there was no clear explanation for the effect of straw mulching on winter wheat grain yield. Chen *et al.* ^10^ indicated that straw mulching increased soil moisture, which in turn improved winter wheat grain yield and water use efficiency. In Morocco, Marbet *et al.* ^11^ found that straw mulching significantly increased winter wheat grain yield. In the same cropping system, conservation tillage and straw mulching significantly boosted yields, improved the use efficiency of limited water resources in arid areas, and lowered carbon emissions from farming^12^. Chen *et al.* ^13^ indicated, however, that in the North China Plain, straw mulching significantly decreased winter wheat grain yield. Straw mulching reduced soil temperature and delayed crop growth and development^14^. Li *et al.* ^15^ showed that straw mulching significantly increased the number of kernel per spike and the 1000-grain weight, but significantly reduced the number of spikes. The net effect was the reduction in winter wheat grain yield. In contrast, Chen *et al.* ^16^ stated that straw mulching of winter wheat not only reduced the number of spikes, but also lowered 1000-kernel weight. Therefore, although straw mulching of winter wheat helps to lower soil water evaporation and carbon emissions, its value as a farming practice is compromised because it lowers crop yields by reducing the number of spikes formed^17^.

At Shandong Agricultural University, China, Yu *et al.* ^18^ proposed a strategy to improve winter wheat yield by implementing wide-precision planting. In conventional-cultivation winter wheat planting, the sowing swath is 3–5 cm, whereas in wide-precision planting, it is 6–8 cm. In addition, seeds are separated from each other in wide-precision sowing as opposed to planting all the seeds in a line in conventional-cultivation planting. The seeding rate is the same for both. Li *et al.* ^19^ showed that grain yield was significantly higher in wide-precision planting than in conventional-cultivation planting. Yield gains in wide-precision planting were attributed primarily to the higher number of spikes relative to those formed in conventional-cultivation planting. Because straw mulching reduces winter wheat grain yield by decreasing the number of spikes, it was proposed that wide-precision planting of winter wheat might compensate for straw mulching yield losses by increasing the number of spikes.

The main determinant of the number of spikes at the maturity stage of winter wheat crop development is the number of tillers^19^. Therefore, the objectives of this study were to determine the (i) effect of wide-precision planting on winter wheat tiller number at different growth stages, (ii) above-ground dry matter accumulation and leaf area index (LAI), and (iii) grain yield and yield composition. By addressing the above questions, it is possible to provide a theoretical basis for the development of a winter wheat planting program in the North China Plain that could reduce both soil water losses and carbon emissions.

## Methods

### Experimental site

**Figure 1:**
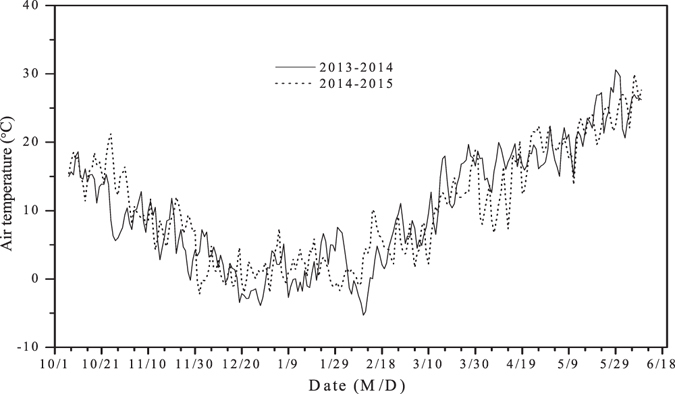
Air temperature in 2013–2014 and 2014–2015 winter wheat growing seasons.

The experiment was conducted at the Experimental Station of the Shandong Agricultural University (36°10“9′N, 117°9“03′E) in the North China Plain during the 2013–2014 and 2014–2015 winter wheat growing seasons. The most widely adopted cropping system in that region is winter wheat and summer maize double cropping system within a year. The area has a temperate continental semi-monsoon climate with an average annual precipitation of 697 mm. Approximately 70% of the annual precipitation occurs from July to September, the summer maize growing season. To obtain high and stable winter wheat grain yields, it is necessary to apply additional irrigation. Mean temperature at the site was 12.9 °C; air temperature in the both growing seasons is presented in Fig. 1. Each experimental plot was 5 m × 5 m. Beds were placed around the plots to prevent soil water runoff. A loam soil was used. Within the top 20 cm of soil, the nitrogen, phosphorus, and potassium levels were 108.1, 16.1, and 92.4 mg/kg, respectively. The field capacity was 32.4%. Previous studies conducted in this region showed that the winter wheat crop realizes reasonable grain yields and uses water efficiently when irrigated to a depth of 120 mm^15, 19–21^. Irrigation at the jointing stage increases tiller number and spike rate, whereas irrigation at the heading stage increases above-ground dry matter accumulation^22^. For these reasons, the plot was irrigated to a depth of 60 mm at both the jointing and heading stages of winter wheat. In the 2013–2014 winter wheat growing season, irrigation was applied on 30 March and 15 April for the jointing and heading stages, respectively. In the 2014–2015 season, irrigation was conducted on 1 April and 19 April. A flow meter was used to control the rate of irrigation water output.

### Experimental Designs

The experiment used a split-plot design. The main plots were planting patterns, i.e., wide-precision planting (W) and conventional-cultivation planting (C). Subplots consisted of two mulching conditions, namely, straw mulching (M) and non-mulching (N). Line spacing in the two planting patterns was 30 cm, but the sowing width in wide-precision planting was 6–8 cm, whereas in conventional-cultivation planting it was 2–3 cm^21^. Mulching material was summer maize straw which was prepared by cutting post-harvest straw into 3–5 cm strips. At the winter wheat seeding stage, straw mulch was manually applied at the rate of 0.6 kg/m^2^. Each treatment was replicated three times. Treatments were randomized within the split plot. Before winter wheat sowing, 19.2 g/m^2^ urea, 26.1 g g/m^2^ diammonium phosphate, and 21.0 g g/m^2^ potassium were applied. At the winter wheat jointing stage, an additional 19.2 g/m^2^ urea was applied along with irrigation. The Jimai 22 variety winter wheat, widely planted in the North China Plain, was hand-sown at a density of 444 plants/m^2^ on October 8, 2013, and October 7, 2014. Thinning was conducted by hand five days after seedling emergence to obtain a final population density of 222 plants/m^2^. The crops were harvested on June 6, 2014 and June 9, 2015, respectively.

### Measurements

#### Soil temperature

In the 2013–2014 winter wheat growing season, at the overwintering, jointing, and milking stages, soil temperature was obtained by time-domain reflectometry (Institute of Agrophysics Polish Academy of Sciences, Lublin, Poland) at the depth of 10 cm at 08:00–09:00.

#### Soil moisture

The volumetric soil moisture content of the cores obtained at every 10.0 cm down to 120.0 cm was measured by a CNC503D neutron moisture meter (Super Energy, Nuclear Technology Ltd., Beijing, China). The soil moisture content of the top 20.0 cm soil layer was measured using the oven-drying method. The soil moisture change was calculated as the difference between the soil moisture content at the sowing stage and the soil moisture content at the maturity stage.

#### Tiller number

In the 2013–2014 and 2014–2015 winter wheat growing seasons, the tiller number was estimated at the overwintering, jointing, and heading stages. At these stages, 50 cm lengths of two rows were selected at random to determine the number of tillers on per square meter.

Tiller extinction rate was defined as follows^23^:

1 $${\rm{Tiller}}\,{\rm{extinction}}\,{\rm{rate}}=\frac{A-B}{A}\times 100 \% $$

In equation (1), A (plants/m^2^) is the tiller number at the jointing stage, and B (plants/m^2^) is the tiller number at the heading stage.

#### Aboveground dry matter accumulation

At the winter wheat maturity stage, 20 representative plants were harvested, and scissors were used to divide the plants into stems, leaves, and spikes. The plant organs were then kept in a drying oven at 105 °C for 20 min, further dried to a constant weight at 80 °C, and then weighed with an electronic balance.

#### Leaf area index

At the winter wheat jointing, heading, and filling stages, 20 representative plants were harvested, and the length and maximum width of each leaf were measured. The leaf area was calculated using the following equation^21^:

2 $${\rm{leaf}}\,{\rm{area}}={\rm{leaf}}\,{\rm{length}}\times {\rm{leaf}}\,{\rm{width}}\times 0.78$$

In equation (2), the leaf length is defined as the distance from leaf base to leaf tip, and the leaf width is defined as the widest part of the leaf.

#### Grain yield

When the winter wheat reached the maturity stage, 2.0-m stretches of two rows were selected at random in each experimental plot and the spike number, 1000-kernel weight, and grain yield were measured. The plants were harvested manually and air-dried. An additional 20 plants were harvested to determine the number of kernel per spike. All of the data in the study were reported as the averages of three consecutive measurements.

### Statistical analysis

Using Microsoft Excel 2007 and SPSS (Statistical Product and Service Solutions) for data processing and statistical analysis, we performed an analysis of variance (ANOVA). Before the ANOVA, data were assessed when necessary. The ANOVA was executed at a significance level of α = 0.05 to determine whether differences existed among treatment means.

## Results

### Soil temperature and soil moisture change

**Figure 2:**
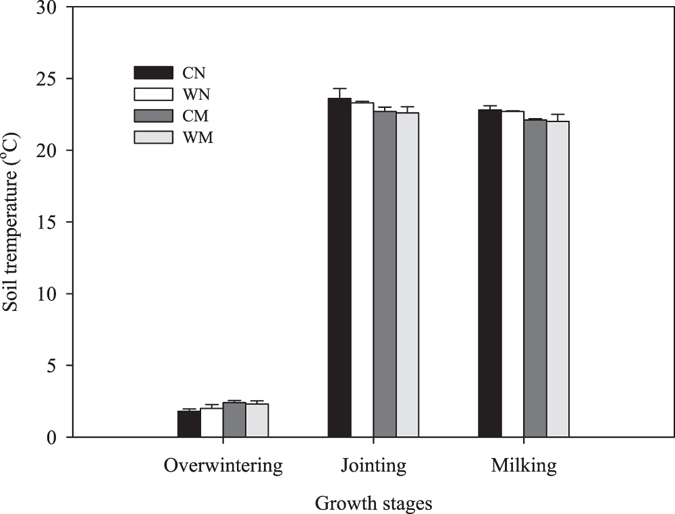
Soil temperature at the depth of 10 cm in 2013–2014 winter wheat growing season. WN represent wide-precision planting with non-mulching, CN represent conventional-cultivation planting with non-mulching, WM represent wide-precision planting with straw mulching, and CM represent conventional-cultivation planting with straw mulching, respectively. Vertical bars are standard errors.

Soil temperature at the depth of 10 cm in the 2013–2014 winter wheat growing season is presented in Fig. 2. During the overwintering, jointing, and milking stages, no differences were found between the wide-precision planting pattern and conventional-cultivation planting pattern. At the overwintering stage, soil temperature was much higher in the mulching treatments than in the non-mulching treatments regardless of whether wide-precision planting or conventional-cultivation planting was used; however, at both the jointing and heading stages, soil temperature was significantly lower in the mulching treatments than in the non-mulching treatments.

**Figure 3:**
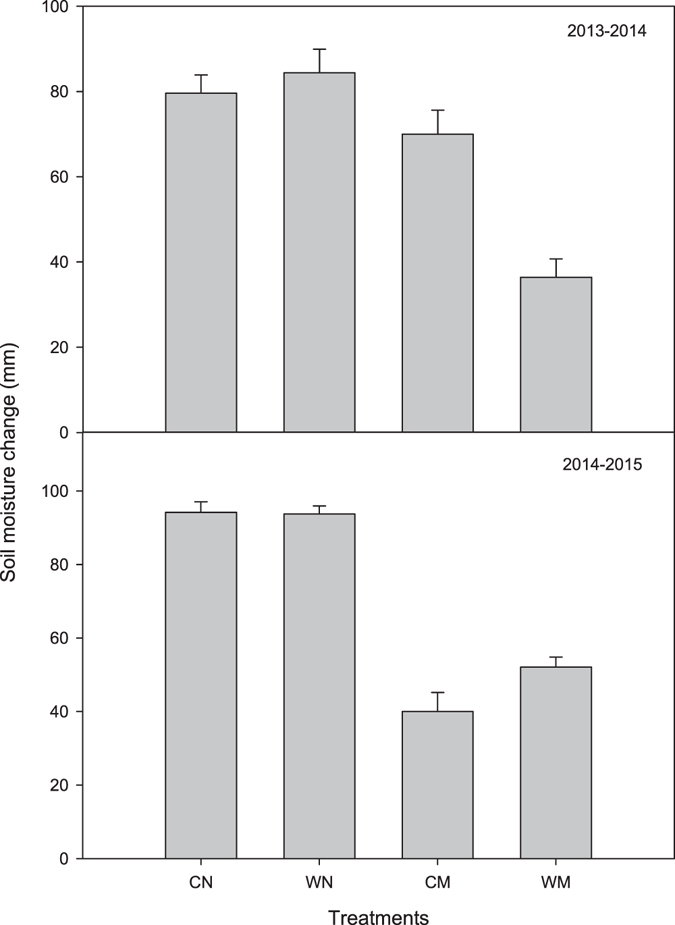
Soil moisture change in 2013–2014 and 2014–2015 winter wheat growing seasons. WN represent wide-precision planting with non-mulching, CN represent conventional-cultivation planting with non-mulching, WM represent wide-precision planting with straw mulching, and CM represent conventional-cultivation planting with straw mulching, respectively. Vertical bars are standard errors.

Soil moisture change in the both growing seasons was determined (Fig. 3); the soil moisture change was significantly lower in the mulching treatments than in the non-mulching treatments regardless of whether wide-precision planting or conventional-cultivation planting was used. In the 2013–2014 winter wheat growing season, soil moisture change was significantly lower by 12.1% in the CM (conventional-cultivation planting with mulching) than in the CN (conventional-cultivation planting with non-mulching) treatment, and lower by 57.3% in the WM (wide-precision planting with mulching) than in the WN (wide-precision planting with non-mulching) treatment. In the 2014–2015 winter wheat growing season, soil moisture change was significantly lower by 57.5% in the CM than in the CN treatment, and by 44.5% in the WM than in the WN treatment.

### Tiller number and quality

**Figure 4:**
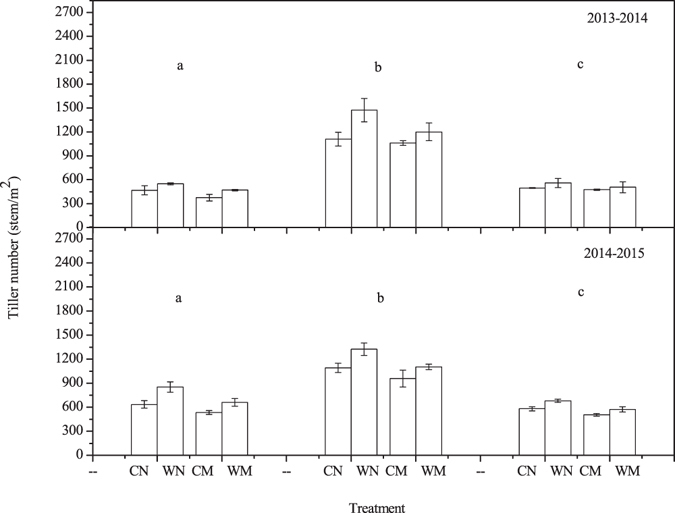
Tillers number at the overwintering stage (**a**), jointing stage (**b**), and heading stage (**c**) in 2013–2014 and 2014–2015 winter wheat growing seasons. WN represent wide-precision planting with non-mulching, CN represent conventional-cultivation planting with non-mulching, WM represent wide-precision planting with straw mulching, and CM represent conventional-cultivation planting with straw mulching, respectively. Vertical bars are standard errors.

Figure 4 shows the tiller number at the overwintering, jointing, and heading stages in the 2013–2014 and the 2014–2015 winter wheat growing seasons. In the 2013–2014 winter wheat growing season, at the overwintering stage, the tiller number was significantly lower in the straw mulching treatments than in the non-mulching treatments regardless of whether wide-precision planting or conventional-cultivation planting was used. Tiller number was significantly higher with wide-precision planting than with conventional-cultivation planting; the highest value occurred in the WN treatment, followed by WM and CN. The lowest value occurred in the CM treatment. Tiller number reached its maximum value at the jointing stage. The tiller number was significantly higher in the WN than in the CN, but no significant difference was found between tiller number for the WM and CM treatments. At the heading stage, tiller die-off lowered numbers relative to those at the jointing stage for all treatments. Tiller number in WN, WM, CN, and CM was reduced by 62.1%, 57.9%, 55.3% and 55.2%, respectively, relative to the jointing stage, and the extinction rate decreased by 12.9% in WN relative to CN and 6.4% more in WM than in CM. Although there were no significant differences between CN and CM, the extinction rate decreased by 4.2% in WM relative to that of WN; hence, there more tillers survived in the WM than in the WN treatment.

Results similar to those observed for the overwintering and jointing stages in the 2013–2014 winter wheat growing season were found in 2014–2015. At the heading stage, tiller number in WN, WM, CN, and CM was reduced by 46.8%, 48.7%, 47.3% and 48.2% respectively relative to the jointing stage; the extinction rate decreased by 16.9% more in WN than in CN and 13.2% more in WM than in CM. In wide-precision planting pattern, the extinction rate decreased by 0.5% more in WM than in WN. Hence, in both growing seasons, in wide-precision planting pattern, more tillers survived in mulching conditions than in non-mulching conditions. As a result, wide-precision planting pattern combined with straw mulching improved tiller quality.

### Aboveground dry matter accumulation

**Figure 5:**
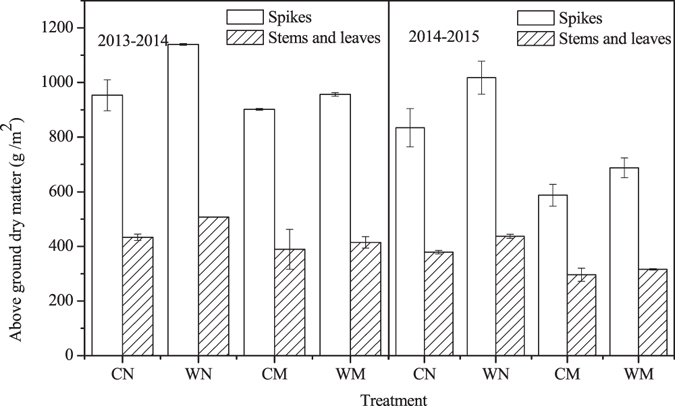
Aboveground dry matter accumulation at the maturity stage in 2013–2014 and 2014–2015 winter wheat growing seasons. WN represent wide-precision planting with non-mulching, CN represent conventional-cultivation planting with non-mulching, WM represent wide-precision planting with straw mulching, and CM represent conventional-cultivation planting with straw mulching, respectively. Vertical bars are standard errors.

For all treatments, the aboveground dry matter accumulations at the winter wheat maturity stage were similar for both growing seasons (Fig. 5). In the 2013–2014 winter wheat growing season, the amount of spike dry matter was 16.1% higher in WN than in WM, and 5.4% higher in CN than in CM. The amount of spike dry matter was 6.0% greater in the WM treatment than in the CM treatment, and 19.5% greater in the WN treatment than in the CN treatment. All of the aforementioned differences were statistically significant. The differences in the amount of dry matter derived from stems and leaves were consistent with those determined for the amounts of dry matter found in the spikes. The amount of dry matter derived from stems and leaves was 18.2% higher in the WN treatment than in the WM treatment, and 10.1% higher in the CN treatment than in the CM treatment. The amount of dry matter derived from stems and leaves was 6.4% higher in the WM treatment than in the CM treatment, and 17.1% greater in the WN treatment than in the CN treatment. All of the aforementioned differences were statistically significant.

In the 2014–2015 winter wheat growing season, the amount of dry matter derived from the spikes was 32.4% greater in the WN treatment than in the WM treatment, and 29.6% greater in the CN treatment than in the CM treatment. The amount of dry matter derived from the spikes was 17.2% higher in the WM treatment than in the CM treatment, and 22.0% higher in the WN treatment than in the CN treatment. All of the aforementioned differences were statistically significant. The differences in the amount of dry matter derived from stems and leaves were consistent with those determined for the amounts of dry matter found in the spikes. The amount of dry matter derived from stems and leaves was 27.7% higher in the WN treatment than in the WM treatment, and 21.8% higher in the CN treatment than in the CM treatment. The amount of dry matter derived from stems and leaves was 6.7% greater in the WM treatment than in the CM treatment, and 15.4% greater in the WN treatment than in the CN treatment. All of the aforementioned differences were statistically significant.

### Leaf area index (LAI)

**Figure 6:**
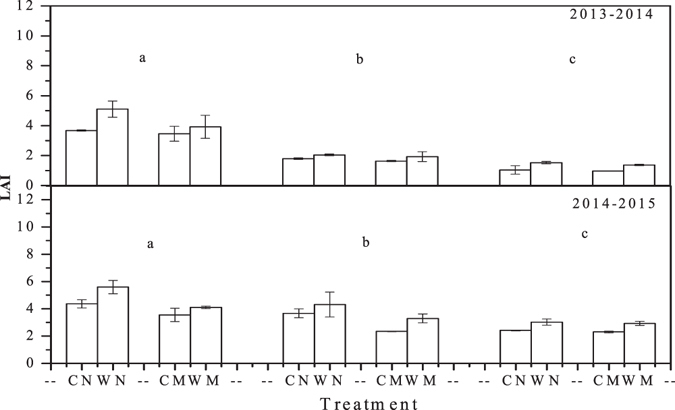
Leaf area index at the jointing (**a**), heading (**b**), and filling (**c**) stages in 2013–2014 and 2014–2015 winter wheat growing seasons. WN represent wide-precision planting with non-mulching, CN represent conventional-cultivation planting with non-mulching, WM represent wide-precision planting with straw mulching, and CM represent conventional-cultivation planting with straw mulching, respectively. Vertical bars are standard errors.

At the jointing stage, LAI was significantly lower in mulching treatments than in non-mulching treatments, regardless of the planting method (Fig. 6). Under non-mulching conditions, the LAI was significantly greater in wide-precision planting than in conventional-cultivation planting, whereas under mulching conditions no significant difference in LAI was found between the planting patterns. The highest LAI was found in the WN treatment, followed by the WM, CN, and CM treatments. At the heading stage, the WN treatment had the highest LAI, followed by the WM, CN, and CM treatments. Therefore, the LAI was significantly higher in wide-precision planting than in conventional-cultivation planting. At the filling stage, the LAI was not significantly different between mulching and non-mulching treatments. Under mulching conditions, the LAI was significantly higher in wide-precision planting than in conventional-cultivation planting.

### Yield and yield compositions

Yield and yield compositions are presented in Table 1. In the 2013–2014 winter wheat growing season, a maximum grain yield of 782.0 g/m^2^ was observed in the WN treatment, whereas a minimum grain yield of 686.7 g/m^2^ was observed in the CM treatment. Grain yield was 5.2% greater in wide-precision planting than in conventional-cultivation planting. The number of spikes was 9.2% higher in wide-precision planting than in conventional-cultivation planting. These differences were statistically significant. The number of spikes and the 1000-grain weight were 15.9% and 4.9% lower, respectively, in mulching treatments than in non-mulching treatments, resulting in an overall 7.8% grain yield reduction in mulching treatments relative to non-mulching treatments. These differences were statistically significant. Grain yield in wide-precision planting was 13.9 g/m^2^ greater than that in conventional-cultivation planting. This result occurred primarily because the number of spikes derived from the wide-precision planting was 39.8 g/m^2^ greater than that of conventional-cultivation planting. These differences were statistically significant.

**Table 1:** Grain yield and yield compositions in 2013–2014 and 2014–2015 winter wheat growing seasons.

Treatments	Spike number (spike/m^2^)	Kernel number per spike (kernel/spike)	1000–kernel weight (g)	Grain yield (g/m^2^)
2013–2014				
By planting				
W	507.9a	38.3a	46.7a	741.3a
C	465.2b	38.2a	46.3a	704.7b
*P value*	*0.1799*	*0.8709*	*0.7208*	*0.1127*
By mulching				
M	528.5a	38.7a	47.7a	752.4a
N	444.6b	37.8a	45.3b	693.7b
*P value*	*0.0012*	*0.3598*	*0.0017*	*0.0063*
Interaction				
WN	551.2a	39.5a	48.0a	782.0a
WM	464.5c	37.2a	45.3b	700.6c
CN	505.7b	37.9a	47.3a	722.7b
CM	424.7d	38.4a	45.3b	686.7d
*P value*	*0.0014*	*0.4384*	*0.0222*	*1.6753*
2014–2015				
By planting				
W	587.5a	41.19a	42.05a	934.75a
C	532.85b	41.22a	41.85a	893.89b
*P value*	*0.0003*	*0.4168*	*0.7196*	*0.0930*
By mulching				
M	632.5a	40.9a	42.8a	945.9a
N	487.8b	41.5a	41.1b	882.8b
*P value*	*0.0000*	*0.9749*	*0.0014*	*0.0027*
Interaction				
WN	661.1a	40.9a	42.8a	965.7a
WM	513.9c	39.8a	41.3b	903.8b
CN	604.0b	39.3a	42.7a	926.1b
CM	461.7d	39.0a	41.0b	861.7c
*P value*	*0.0000*	*0.6207*	*0.0037*	*0.0007*

WN represent wide-precision planting with non-mulching, CN represent conventional-cultivation planting with non-mulching, WM represent wide-precision planting with straw mulching, and CM represent conventional-cultivation planting with straw mulching, respectively. In each growing season, values followed by different letters are significantly (P < 0.05) different among treatments. The in italics are the P value of the significance. When P < 0.05 means “significantly different”, and when P > 0.05 means “not significantly different”.

In the 2014–2015 winter wheat growing season, a maximum grain yield of 965.7 g/m^2^ was observed in the WN treatment, whereas a minimum grain yield of 861.7 g/m^2^ was observed in the CM treatment. Grain yield was 4.6% greater in wide-precision planting than in conventional-cultivation planting. The number of spikes was 10.3% higher in wide-precision planting than in conventional-cultivation planting. These differences were statistically significant. The number of spikes and the 1000-grain weight were 22.9% and 3.8% lower, respectively, in mulching treatments than in non-mulching treatments, resulting in an overall 6.7% grain yield reduction in mulching treatments relative to non-mulching treatments. These differences were statistically significant. Grain yield in wide-precision planting was 42.1 g/m^2^ greater than that in conventional-cultivation planting. This result occurred primarily because the number of spikes derived from the wide-precision planting was 52.2 g/m^2^ greater than that of conventional-cultivation planting. These differences were statistically significant.

## Discussion

The study showed that whether wide-precision planting or conventional-cultivation planting was adopted, grain yield decreased under straw mulching, which was mainly caused by the reduction in spike number and 1000-kernel weight. This result may have occurred because of the following reasons: (i) temperature effect: In this study, at jointing and milking stages, soil temperature at the depth of 10 cm was much lower in mulching treatments than in non-mulching treatments regardless of whether wide-precision planting or conventional-cultivation planting was used. From 0 °C to 40 °C, lower soil temperature hinders root system expansion^15^. Chen *et al.* ^13^ found that after straw mulching, the overall effects of mulch on soil temperature were reduced diurnal and nocturnal soil temperatures. After recovery, when winter wheat reaches the rapid growing period, the delay in development decelerated dry matter accumulation. Thus, the final grain yield of mulched winter wheat was affected. However, in the late growth stage, compared with non-mulching treatments, vegetative organ growth in mulching treatments was very rapid^15^. (ii) Allelopathy effects: Allelopathic expression during some growth periods induced a partial correlation effect for some important agronomic characters that affected wheat yield. Allelopathic expression during the germination and seeding-filling stages in different wheat accessions had a detrimental effect on plant height. Allelopathic potentials had significant detrimental effects on spikelet number during the tillering and flowering stages, on 1000-kernel weight during greening and stem elongation, and on tillering during the seedling and flowering stages^24, 25^. Yang *et al.* ^26^ indicated that the allelopathic effect of maize straw on wheat seedlings was the strongest, leading to 60.8% reduction in wheat biomass. To explore the biochemical effects on the decrease in winter wheat grain yield, a method for the use of other seasonal straws to replace maize straw is needed in future research.

The grain yield of both planting patterns was much lower in mulching treatments than in non-mulching treatments. The improvement of grain yield under straw mulching conditions required immediate attention. Li *et al.* ^15^ showed that furrow-planting combined with deficit irrigation changed the vertical distribution of LAI and significantly increased the LAI at 60–80 cm above the ground surface; functional leaves and spikes were concentrated in this layer, and hence radiation use efficiency and grain yield were improved. Zhao *et al.* ^21^ found that wide-precision planting could improve solar energy utilization under deficit irrigation, and hence benefit dry matter accumulation for spikes. Because wide-precision planting changes the sowing width from 3–5 cm to 6–8 cm, this may not affect the value of LAI but rather its vertical distribution. Under mulching conditions, this maybe an important reason why aboveground dry matter accumulation was much higher in wide-precision planting than in conventional-cultivation planting; however, this topic needs further exploration.

Straw mulching reduced winter wheat grain yield by decreasing the number of spikes. Compared to conventional-cultivation planting, wide-precision planting could help to compensate for grain yield losses, mainly because wide-precision planting increased winter wheat tiller number, and reduced tiller die-off. In field conditions, the higher LAI value, the lower the soil evaporation rate^15^. Thus, soil moisture content in the upper soil layer maybe considerably higher in wide-precision planting fields than in conventional-cultivation planting fields. This could be why tiller die-off was reduced by wide-precision planting compared to conventional-cultivation planting. As a result, wide-precision planting could reduce the adverse effects of straw mulching on spike number. However, with straw mulching, wide-precision planting reduced the production losses to some extent, but yield was still significantly lower than that of non-mulching treatments. Regarding yield composition, only spike number significantly increased, and neither kernel number per spike nor 1000-kernel weight were significantly affected. Therefore, to enhance the compensation effect further, it is extremely important to find a way to increase spike number, kernel number per spike, and 1000-kernel weight simultaneously.

Lang *et al.* ^27^ indicated postponing jointing irrigation could further reduce the death of tillers in wide-precision planting; as a result, spike number, kernel number per spike, and grain yield would increase significantly. Therefore, under straw mulching conditions, wide-precision planting through delayed jointing irrigation could result in both increased grain yield and water use efficiency simultaneously. Under field conditions, the essence of water use efficiency is the coupling effect of carbon absorption and evapotranspiration^28^. In the North China Plain, because water resources are limited, increasing water use efficiency is the only way to ensure sustainable development of winter wheat production. Therefore, in wide-precision planting, balancing carbon absorption and evapotranspiration under the straw mulching and deficit irrigation is important to ensure sustainable development of winter wheat production in the North China Plain, which will aid in the establishment of a water-saving and high-yielding planting pattern.

With straw mulching, a compensatory effect of winter wheat grain yield reduction was observed in wide-precision planting. Straw mulching can decrease soil CO_2_ emissions^12, 29, 30^, increase soil organic matter^9, 31^, enhance rapid available phosphorus and potassium^31^, and improve nitrogen use efficiency^32^. Hence, wide-precision planting combined with straw mulching may be a new environment-friendly method of increasing winter wheat yield.

## Conclusion

Straw mulch significantly reduced winter wheat grain yield, mainly by lowering the tiller number and dry matter accumulation at late growth stages. Tiller number was significant higher in wide-precision planting than in conventional-cultivation planting. Wide-precision planting under straw mulching conditions compensated for winter wheat grain yield losses primarily by significantly increasing the spike number.
